# Clinical Large Language Model Evaluation by Expert Review (CLEVER): Framework Development and Validation

**DOI:** 10.2196/72153

**Published:** 2025-12-04

**Authors:** Veysel Kocaman, Mustafa Aytuğ Kaya, Andrei Marian Feier, David Talby

**Affiliations:** 1 John Snow Labs Inc, 16192 Coastal Highway, Lewes, DE, 19958, United States, +1 (302) 786-5227; 2 Computational Sciences and Informatics (CSI), George Mason University, Fairfax, VA, United States

**Keywords:** large language models, medical LLMs, evaluation framework, clinical relevance, factuality, natural language processing, generative AI, NLP, artificial intelligence

## Abstract

**Background:**

The proliferation of both general purpose and health care–specific large language models (LLMs) has intensified the challenge of effectively evaluating and comparing them. Data contamination plagues the validity of public benchmarks, self-preference distorts LLM-as-a-judge approaches, and there is a gap between the tasks used to test models and those used in clinical practice.

**Objective:**

In response, we propose CLEVER (Clinical Large Language Model Evaluation–Expert Review), a methodology for blind, randomized, preference-based evaluation by practicing medical doctors on specific tasks.

**Methods:**

We demonstrate the methodology by comparing GPT-4o (OpenAI) against 2 health care–specific LLMs, with 8 billion and 70 billion parameters, over 3 tasks: clinical text summarization, clinical information extraction, and question answering on biomedical research.

**Results:**

Medical doctors prefer the medical model–small LLM trained by John Snow Labs over GPT-4o 45% to 92% more often on the dimensions of factuality, clinical relevance, and conciseness.

**Conclusions:**

The models show comparable performance on open-ended medical question answering, suggesting that health care–specific LLMs can outperform much larger general purpose LLMs in tasks that require understanding of clinical context. We test the validity of CLEVER evaluations by conducting interannotator agreement, interclass correlation, and washout period analysis.

## Introduction

### Challenges in Trustworthy Large Language Model Evaluation

The emergence of large language models (LLMs) has transformed natural language processing (NLP) across numerous fields. Recent innovations, particularly LoRA (Low-Rank Adaptation) [1], have markedly improved these models’ accessibility and adaptability. LoRA’s approach of minimizing trainable parameters enables the efficient adaptation of large models to specific domains with limited computational resources, often yielding results that match or exceed those of full fine-tuning.

This parameter-efficient method not only expedites the adaptation process but also helps prevent overfitting, especially when dealing with scarce domain-specific data. Consequently, these refined, more compact models can potentially outperform their larger, general purpose counterparts in specialized areas without encountering issues such as catastrophic forgetting [2]. The advent of such techniques has democratized LLM deployment, empowering a wider array of researchers and organizations to harness these powerful tools without requiring substantial computational infrastructure. This accessibility has opened new avenues for innovation and application across diverse domains, fostering a more inclusive landscape in artificial intelligence (AI) research and development.

However, while the ability to create domain-specific LLMs has expanded, the challenge of effectively evaluating their performance remains. LLMs evaluation is challenging due to the complexities of scoring and the difficulties associated with reproducibility. Current evaluation methods include multiple-choice questions, heuristics, and string matching, using other language models for assessments (LLM as a judge), and hiring human annotators. While multiple-choice evaluations are a cost-effective and straightforward approach, they do not accurately reflect real-world performance and often oversimplify the evaluation process. This method fails to adequately assess a model’s capability to generate longer sequences or engage in chain-of-thought reasoning. Furthermore, it is not compatible with real-world scenarios where open-ended responses are required, as users expect models to generate answers rather than select from given options [3].

Additionally, while it is possible to measure log likelihood when using multiple-choice questions, this method only measures the truthfulness of the only token generated by the model and cannot provide an in-depth evaluation. The limitations of current evaluation methods become even more pronounced when considering the need for sophisticated techniques capable of assessing linguistic accuracy, relevance, and potential impact in specialized fields such as medicine, where errors can have serious consequences. Perplexity is another metric that can be used to measure a model’s predictive capabilities by assessing how well it fits a given distribution. However, perplexity may not be suitable for instruction-tuned models and can be distanced from actual model quality in real-world generation scenarios. Moreover, both log likelihood and perplexity are tokenizer-specific metrics that require careful tuning and consideration of implementation details [4].

In real-world scenarios, text generation remains a crucial area of focus, especially for applications such as summarization and open-ended question answering (QA). However, evaluating free-form text generation is inherently difficult. Typically, evaluated models are forced to resort to heuristics for answer extraction to make them measurable, leading to outputs that can be partially correct or follow specific patterns without fully addressing the intended task. This creates challenges in determining the quality of responses. Another significant concern in evaluations is reproducibility. Ideally, published results should provide sufficient detail for others to replicate findings within a narrow margin of error. However, reproducibility can be compromised by standard evaluation libraries and test sets that may not adequately reflect real-world scenarios.

At first glance, one may assume that LLMs can effectively tackle the challenges associated with evaluating open text generation, given their capacity to simulate human evaluation. However, this approach introduces a potential circular validation issue, as the judging LLM must also be evaluated for domain accuracy. If an LLM were adequately trained for specialized domain evaluations, it would be more advantageous to apply it directly to these tasks rather than using it as a judge. Some efforts have been made to use multiple LLM judges to boost capabilities, such as in the study by Badshah and Sajjad [5]; however, it is predominantly confined to question-answering datasets, where judge LLMs provide binary true or false verdicts on candidate LLM outputs on generic tasks, leaving a gap in addressing the complexities of real-life medical scenarios and the open-ended nature of text generation. Moreover, these evaluations are generally benchmarked against human evaluations, underpinning the idea of relying on human judgment for complex tasks, since it is generally acknowledged that LLMs cannot surpass the performance of subject-matter experts in tasks that necessitate subjective reasoning. Despite the higher costs and scalability challenges associated with human evaluations, they are often regarded as the gold standard due to their capacity to provide nuanced assessments and an in-depth understanding of subject matter.

The authors of this paper acknowledge that while human evaluation is often considered the gold standard, its effectiveness is contingent upon the evaluators possessing domain-specific expertise. LLMs expand across various applications—from consumer electronics to heavy machinery—the need for tailored evaluation methods becomes increasingly critical. Each domain-specific LLM should undergo unique evaluation processes, particularly in sensitive fields such as health care, where accuracy and reliability are paramount. Medical evaluations must be conducted by medical experts who can assess the clinical relevance and factual accuracy of LLM outputs, ensuring that these models meet the stringent requirements of real-world medical practice. This approach is essential not only to validate the performance of LLMs but also to address the complexities and nuances inherent in specialized domains, thereby fostering trust and effectiveness in their deployment.

In light of the aforementioned principles, a review of current medical LLM evaluation methods reveals significant shortcomings. Existing evaluations have primarily relied on multiple-choice examination questions and synthetic datasets. While these methods are useful for assessing medical knowledge representation and reasoning, they fail to capture the complexities of real-world clinical applications. Critical aspects such as comprehension of complex patient cases from semistructured documents, understanding medical jargon and abbreviations, information reformulation for tasks such as summarization, and open-ended QA are often overlooked. Recent studies have also highlighted issues with data contamination in popular benchmarks such as MMLU (Massive Multitask Language Understanding) [6], raising concerns about their validity. Notably, most evaluations have not used clinical text datasets that differ significantly from quiz questions in terms of structure, language, and length. Although some benchmarks such as DR.BENCH (Diagnostic Reasoning Benchmark) [7] focus on clinical documents, they primarily consist of traditional encoder model tasks, limiting their suitability for comprehensive LLM evaluation. Recent attempts to create datasets from electronic health records or fictive patient cases have not yet been used to evaluate biomedical LLMs. These limitations underscore the need for a more comprehensive and clinically relevant evaluation framework for medical LLMs [8].

Similarly, in a parallel study, Bedi et al [9] conducted a systematic review of 519 studies on LLMs in health care, revealing significant gaps in current evaluation practices. Only 5% of studies used real patient care data, with most focusing on medical knowledge tasks (44.5% on medical licensing examination questions) and diagnostic tasks (19.5%). In terms of NLP and natural language understanding tasks, QA dominated (84.2%), while summarization (8.9%) and conversational dialog (3.3%) were infrequent. The review also identified several key shortcomings in existing evaluations: there was an overemphasis on accuracy metrics, with 95.4% of studies using accuracy as the primary evaluation dimension, which led to the introduction of “factuality” as an additional evaluation dimension to assess the truthfulness of LLM outputs by the authors. Moreover, medical specialties were unevenly represented, with most studies focusing on generic health care applications [9].

Another challenge roots from the widespread use of mainstream benchmarks for evaluating LLMs, posing a significant risk of benchmark leakage. Models may have been exposed to evaluation data during training, which in turn can lead to inflated metrics and unrealistic test outcomes, favoring larger models trained on extensive corpora. Recent research has shown that many open-source LLMs have indeed been exposed to benchmark datasets during training, resulting in potentially unreliable performance scores.

On another note, research by Ni et al [10] in their paper “Training on the Benchmark Is Not All You Need” reveals that many prominent open-source LLMs have been exposed to benchmark datasets during their training process. This exposure can lead to inflated and potentially unreliable performance scores, obscuring the true capabilities of these models and making it challenging to accurately assess their performance on real-world tasks outside the benchmark context. In a parallel study, Deng et al [11] found that ChatGPT (OpenAI) was able to guess missing options by 57% in the MMLU benchmark, with the model demonstrating a remarkable ability to predict masked choices in test sets, raising significant concerns about potential data contamination in modern evaluation benchmarks.

Similarly, Oren et al [12] proved in a study that test set contamination in language models can be detected by analyzing the likelihood of canonical orderings compared to shuffled examples, demonstrating this method’s effectiveness even in models as small as 1.4 billion parameters and on test sets of only 1000 examples. AI2 ARC on Mistral (Mistral AI), which does return a low *P* value of .001, could suggest some contamination. “Benchmarking Benchmark Leakage in Large Language Models” created a unified methodology from atomic detection metrics [13].

Using a unique dataset for evaluating LLMs is essential for obtaining accurate and relevant performance insights tailored to specific domains. This approach minimizes bias from pre-existing benchmarks that models may have encountered during training, ensuring a fair assessment of their true capabilities. Additionally, a custom dataset allows for the identification of domain-specific challenges, leading to more effective implementations and improvements in real-world applications. By adhering to these principles, we aim to provide a more robust and meaningful evaluation of LLMs in the medical domain, avoiding the pitfalls highlighted by the Goodhart law: “When a measure becomes a target, it ceases to be a good measure” [14]. This adage is particularly relevant in the context of LLM evaluation, where mainstream benchmarks on known datasets carry a high risk of having been consumed during model training, which can create inflated metrics and unrealistic test outcomes, favoring larger models trained on extensive corpora.

### Introducing CLEVER

#### Overview

Recognizing these limitations and the unique requirements of medical LLMs, we developed a comprehensive evaluation framework, CLEVER (Clinical Large Language Model Evaluation–Expert Review), specifically tailored for assessing LLM performance in medical applications.

#### Assembling a Panel of Medical Experts

We formed a diverse panel of medical experts from various specialties, including internal medicine, oncology, and neurology. This multidisciplinary approach ensured that our evaluation process incorporated a wide range of clinical perspectives and expertise. The involvement of these specialists, who also possess extensive knowledge of NLP and practical experience in its application, was crucial for assessing the clinical relevance and factual accuracy of the LLM-generated outputs in the context of real-world medical practice.

#### Assessment of LLM Performance Across Key NLP Tasks

To effectively evaluate the capabilities of the language models, we conducted a thorough assessment across 4 critical medical NLP tasks: medical summarization, QA in clinical notes, biomedical research, and open-ended QA (closed-book) in medical context. Each task was selected based on its relevance to clinical workflows and its potential impact on patient care. Our evaluation framework was grounded in 3 fundamental pillars: factuality, which assesses the accuracy of information; clinical relevance, which evaluates the applicability of the information to real-world scenarios; and conciseness, which measures how effectively the models convey necessary information without unnecessary verbosity.

#### Mitigating Data Contamination Issues

To address concerns regarding data contamination and ensure an unbiased evaluation, we created 500 novel clinical test cases specifically tailored for LLM tasks from scratch, paying specific attention to avoid any overlap with existing benchmark datasets that may have been used during model training. We reserved 100 test cases for interannotator agreement (IAA) analysis to be evaluated by subject-matter expert physicians. All clinical test cases were created from scratch, guided by established clinical practice guidelines and real-world medical documentation formats (eg, discharge summaries, pathology reports, and research abstracts). Medical experts constructed cases to resemble authentic clinical scenarios while explicitly avoiding overlap with existing benchmarks or copyrighted material.

#### Dataset and Test Cases

Topics were chosen to reflect a broad spectrum of medical specialties, including internal medicine, oncology, and neurology, ensuring diversity of subject matter and clinical complexity.

#### Guidelines for Test Case Creation

All test cases were created de novo to ensure originality, clinical authenticity, and fairness in evaluation. The design process adhered closely to established clinical practice guidelines and incorporated the structure and style of real-world medical documentation formats, such as discharge summaries, pathology reports, and research abstracts. To prevent bias and inflated performance metrics, explicit care was taken to exclude any overlap with existing benchmark datasets (eg, MMLU and MedQA) or copyrighted materials. By constructing the cases from scratch, the dataset was purpose-built to mitigate risks of data leakage while providing an unbiased foundation for evaluating LLM performance in clinically realistic contexts.

#### Expert-Driven Design and Validation

Test case development followed a rigorous, expert-driven process to ensure clinical accuracy, realism, and task appropriateness. Initial drafts were created by medical doctors with expertise across multiple specialties, drawing on deidentified electronic health records, clinical case reports, and peer-reviewed biomedical literature. Each case was subsequently reviewed by at least 2 additional physicians not involved in drafting, who evaluated the content for accuracy, clarity, and suitability for the intended task, such as summarization or focused QA. Feedback from these reviews guided iterative refinements until consensus was reached among all reviewers. This multistep process ensured that the final dataset was both clinically valid and methodologically rigorous, providing high-quality test material for evaluating LLM performance in real-world medical contexts.

#### Composition of the Test Set

The test set was designed to comprehensively evaluate medical NLP performance by encompassing a balanced range of narrative and structured items across 4 task categories. A total of 500 cases were developed, distributed in roughly equal proportions (approximately 125 per category) among clinical summarization (CS), clinical information extraction, biomedical research QA, and open-ended medical QA. CS tasks required condensing complex patient cases from semistructured narratives, while information extraction focused on answering diagnostic or management questions from patient notes (eg, “What were the bone marrow smear findings?”). Biomedical research QA tasks involved interpreting abstracts and short scientific reports, and open-ended medical QA items assessed broader medical knowledge in a closed-book setting. Input length and complexity were intentionally varied across cases to reflect the heterogeneity of challenges encountered in real-world medical practice, from short diagnostic prompts to extended case narratives requiring integrative reasoning.

#### Ensuring Scientific Rigor

To uphold scientific rigor throughout the evaluation process, we used a randomized, blind experimental design incorporating multiple comparative tests to minimize bias and enhance reliability. Evaluators were blinded to the source of each response, ensuring they were unaware of which model had generated the outputs under assessment. To further validate the integrity of the results, several measures were implemented, including IAA analysis to assess consistency among expert raters, intraclass correlation coefficient (ICC) calculations to quantify reliability across evaluators, and washout period assessments to evaluate the stability of model performance over time, thereby offering insights into reproducibility.

For IAA analysis, 100 cases were selected from the full dataset using random stratified sampling. This approach ensured proportional representation across all 4 task categories, coverage of the multiple medical specialties included in the dataset, and incorporation of diverse case types, ranging from simple to complex and short to long scenarios. This sampling strategy preserved the heterogeneity of the evaluation dataset and prevented bias toward any specific task or medical specialty.

Blinding and randomization procedures were applied during evaluation to eliminate potential sources of bias. All test cases were stripped of identifiers and randomized in order before being presented to both the models and the human experts. Through this rigorous, multilayered process, the test cases remained novel, free from contamination, clinically authentic, and representative of the practical challenges encountered in medical NLP—ultimately providing a robust foundation for the fair and meaningful evaluation of LLM performance.

This study presents the results of applying CLEVER to compare GPT-4o [15], trained by OpenAI, with 2 health care–specific LLMs trained by John Snow Labs: medical model–small (MedS) [16], which has 8 billion parameters, and medical model–medium (MedM), which has 70 billion parameters. The results demonstrated that after fine-tuning on a domain-specific dataset, these specialized LLMs outperformed GPT-4o in the measured tasks, notably using a model with <10 billion parameters that can be deployed on-premise. These findings validate our proof of concept for evaluations, confirming that our methodology is effective. Furthermore, they support the theory that smaller models trained with domain-specific data can indeed surpass larger models, a hypothesis that has been proven through our rigorous benchmarking process.

Through a randomized blind study conducted with medical doctors as evaluators, we assessed LLM performance based on 3 essential dimensions—factuality, clinical relevance, and conciseness. This approach aligned with best practices in clinical research and acknowledged the inherent complexity of medical information, necessitating comprehensive evaluation metrics. Our CLEVER methodology aimed to transform these theoretical insights into actionable practices within health care. By providing a structured framework for evaluation, CLEVER not only facilitated the effective integration of LLMs into clinical workflows but also highlighted critical areas for enhancement, ensuring that these models could reliably support health care professionals in delivering high-quality patient care.

## Methods

### Medical Experts

Medical professionals are essential for evaluating medical LLMs due to their superior accuracy, domain-specific expertise, and ability to assess potential harm. Research by Avnat et al [17] demonstrates that medical experts significantly outperform LLMs in diagnostic accuracy and clinical decision-making, with professionals achieving 82.3% accuracy compared to 64.3% for Claude3 (ANTHROPIC PBC) and 55.8% for GPT-4 (OpenAI) on identical medical questions.

This substantial performance gap underscores the critical need for expert evaluation to ensure patient safety. Furthermore, medical professionals possess deep domain-specific knowledge that allows them to identify subtle nuances, context-specific information, and potential errors that may elude nonexpert evaluators or automated systems. Expert qualifications enable them to assess the potential for harm in LLM outputs, identifying dangerous advice, misinformation, or inappropriate recommendations that could pose risks to patient health if implemented in clinical settings. Consequently, the involvement of medical professionals in evaluating medical LLMs is crucial for maintaining high standards of accuracy, reliability, and safety in health care applications.

For our research, we convened a diverse panel of medical experts with combined expertise in clinical practice and medical AI solutions. The board included:

- A professor of human pathophysiology (MD, PhD) with 25 years of clinical experience and 2 years in AI solutions
- A neurologist (MD) with 1 year of clinical practice and 3 years in AI solutions
- An ECFMG (Educational Commission for Foreign Medical Graduates)–certified hematologist-oncologist (MD) with 3 years of clinical experience and 3 years in AI solutions

This multidisciplinary team brought deep medical knowledge and AI expertise to the evaluation. All physicians were externally hired with no prior exposure to our models or training data, ensuring unbiased assessments. Their sole role was to prepare evaluation data and objectively evaluate model outputs, independent of model familiarity. This approach guarantees that the evaluation reflects an accurate measure of model performance on unseen data.

### Evaluation Rubrics

In our study, we developed a comprehensive evaluation framework for assessing the performance of LLMs in medical contexts. This framework is built upon 3 key rubrics: factuality, clinical relevance, and conciseness, which were carefully selected to address the unique challenges and requirements of medical information processing and dissemination.

The first rubric, factuality, can be defined as the extent to which the information provided is accurate, truthful, and consistent with established medical knowledge. It means the response is free from factual errors or misleading content that could negatively impact clinical understanding or decisions. Factuality focuses on the accuracy and correctness of the LLM-generated answers. This criterion is of paramount importance in the medical field, where misinformation can have severe consequences. Evaluators were instructed to assess the veracity of the information provided, drawing upon their extensive medical knowledge and referencing current literature and established clinical guidelines.

The assessment of factuality not only considers the presence of correct information but also the absence of false or misleading statements. This approach aligns with recent research [18-21] emphasizing the critical nature of factual consistency in medical AI applications.

Clinical relevance, the second rubric, is defined as the degree to which the information is meaningful and useful in a clinical context. It reflects how well the response supports health care providers in making informed medical decisions and addresses relevant clinical issues. This rubric evaluates the applicability and utility of the LLM-generated information in real-world clinical settings. This criterion addresses a crucial aspect of medical AI: the ability to provide actionable insights that can inform patient care and clinical decision-making. Evaluators were tasked with assessing how well the LLM’s responses addressed the specific medical questions or tasks presented, and to what extent the information could be practically applied in clinical scenarios. This rubric acknowledges that even factually correct information may not always be clinically useful, a distinction that is vital in the context of medical practice.

The third rubric, conciseness, is defined as the ability to communicate necessary information clearly and succinctly, avoiding unnecessary verbosity or jargon. It emphasizes delivering complex medical information in a way that is easy to understand and quickly digestible for efficient application in clinical practice. Conciseness assesses the LLM’s ability to convey necessary information succinctly and clearly. In the fast-paced environment of clinical practice, the ability to quickly digest and apply information is crucial. Evaluators were instructed to consider how effectively the LLM summarized complex medical concepts without sacrificing essential details. This rubric also takes into account the clarity of expression and the avoidance of unnecessary jargon or verbosity. The importance of conciseness in medical communication has been highlighted in several studies [22-25], noting its impact on information retention and application in clinical settings.

### Tasks

The 500 cases were distributed across the 4 task categories: CS, clinical information extraction (QA from notes), biomedical research QA, and open-ended medical QA. Each category was represented in roughly balanced proportions (approximately one-quarter of the dataset per task type), with variation in input length and complexity to reflect the range of challenges encountered in real clinical practice.

CS is a critical task in health care that involves distilling and synthesizing complex medical information into concise, accurate, and actionable summaries. CS produced by a well-adapted LLM can match the expertise of medical professionals [26], enabling health care providers to efficiently interpret patient information and make informed decisions [27].

One of the focal points of CS evaluation is information synthesis, which tests the models’ ability to extract and combine relevant details from diverse sources, such as electronic health records, laboratory results, and clinical notes, which plays a vital role in creating comprehensive summaries reflecting the complexity of patient cases.

Effective CS requires a deep understanding of domain-specific knowledge, including medical terminology, procedures, and the relationships between various health factors. Language models must demonstrate proficiency in these areas to generate summaries that are not only accurate but also clinically relevant. The ability to condense extensive medical histories into brief yet comprehensive narratives highlights the models’ effectiveness in prioritizing the most pertinent information for clinical decision-making.

Additionally, effective CS often involves understanding the chronology of medical events and their implications for patient care, further underscoring the need for temporal reasoning capabilities within these models. Furthermore, evaluating how well models perform across different medical specialties—from general practice to specialized fields such as oncology or neurology—can provide insights into their adaptability and robustness in diverse clinical contexts.

Clinical QA tasks are essential for evaluating language models in the medical domain. This task involves answering specific clinical questions based on the provided medical context. It assesses the model’s ability to extract relevant clinical information and apply it effectively to answer focused inquiries. For example, questions such as “Given the clinical note, what were the bone marrow smear findings?” or “Given the surgical note, what procedures did the patient undergo?” require the model to interpret complex clinical data accurately. Additionally, questions such as “Based on the provided case report, did Anlotinib benefit the patient?” test the model’s understanding of treatment efficacy and patient outcomes. The ability to answer these questions demonstrates a model’s comprehension of medical terminology, procedural knowledge, and clinical reasoning, making it a vital evaluation criterion for language models intended for use in health care settings.

Biomedical research QA tasks focus on evaluating language models’ capabilities in interpreting scientific literature and research findings. This task requires models to answer questions related to biomedical studies, such as “Given the report, what biomarkers are commonly negative in acute promyelocytic leukemia (APL) cases?” or “Given the note, why is chemotherapy the main treatment used in triple-negative breast cancer (TNBC) patients?” These inquiries test the model’s ability to synthesize information from research articles and clinical guidelines while demonstrating an understanding of disease mechanisms and treatment rationales. Furthermore, questions such as “Given the article, what is serum neurofilament light chain (sNFL) used for?” assess the model’s knowledge of specific biomarkers and their applications in clinical practice. By evaluating models on biomedical research QA tasks, researchers can gauge their proficiency in handling complex medical information and their potential utility in supporting health care professionals with evidence-based decision-making.

Open-ended QA tasks evaluate language models’ abilities to provide comprehensive responses to broader medical inquiries that may not have straightforward answers. This task encompasses a wide range of questions, such as “What biopsy techniques confirm malignant bone tumors?” or “Are there any favorable mutations in acute lymphoblastic leukemia?” These types of questions require models to integrate knowledge from various medical domains and provide well-rounded answers based on existing literature and clinical guidelines. Additionally, inquiries such as “Are there any over-the-counter sleep aids that contain barbiturates?” challenge models to synthesize information about pharmacology and regulations surrounding medications. The open-ended nature of these questions tests a model’s depth of understanding and its ability to navigate complex topics while delivering accurate and relevant information.

### Test Setup

We conducted randomized blind evaluations to mitigate potential bias in our assessments, particularly because the medical evaluators are employees of our organization and may be influenced by familiar patterns. Shuffling the order of model responses for each question in comparative evaluations, we aimed to reduce various forms of bias, such as order bias and expectation bias, while also preventing evaluators from memorizing specific models’ styles. By enhancing objectivity and reliability in the assessment process, we compelled evaluators to judge each response solely on its merits without knowledge of its source. Furthermore, by eliminating external cues and preconceptions, we facilitated a fairer comparison between models and revealed genuine preferences, increasing the statistical validity of our results. Ultimately, shuffling model responses contributes to a more robust and trustworthy evaluation of the relative performance of different language models, ensuring that the assessment is based on the quality of the outputs rather than extraneous factors.

Our preferred method for testing model success was pairwise comparison between GPT-4o, MedS, and MedM. Pairwise comparison offers numerous advantages in evaluating complex options, particularly in the context of language model assessment. This method simplifies the evaluation process by breaking it down into manageable comparisons between 2 options at a time, reducing cognitive load and making it easier for evaluators to make decisions. It is especially useful for long lists of items and mimics real-life decision-making processes, potentially leading to more accurate research results. Pairwise comparison can generate quantitative results from qualitative data, handle incomplete comparison data, and minimize biases through randomization. In the context of blind testing language models, this approach allows evaluators to focus on the content of each response without being influenced by knowledge of which model produced it. This leads to a more objective and statistically valid assessment of the models’ relative performance. The method’s simplicity, flexibility, and ability to reduce bias make it an excellent choice for comparing language model outputs, ensuring a fair and thorough evaluation process. A recent study by Liu et al [28] justifies our pairwise comparison approach, demonstrating that pairwise preference methods inspired by reinforcement learning from human feedback can leverage LLMs’ inherent ranking abilities to produce more robust, transitive, and efficient evaluations that align better with human judgments.

We augmented the pairwise comparison method with the inclusion of 4 options (model A vs model B, neutral, and none) to provide several advantages and reflect a nuanced understanding of the complexities involved in comparing language model outputs. First, our augmented comparison allows evaluators to accurately indicate their preference when 1 model clearly outperforms others while accommodating scenarios where each model might excel in different aspects. The “neutral” option recognizes that in some cases, the outputs from different models may be very similar in quality or content, without a clear advantage, thus preventing forced choices when differences are negligible and leading to more accurate overall results. Additionally, the “none” option is crucial as it enables evaluators to indicate when none of the responses meet the required standards of factuality, clinical relevance, or conciseness, which is particularly important in medical contexts where accuracy and relevance are paramount. This 4-option framework helps prevent false positives; without the “none” option, evaluators might feel pressured to select the “least bad” option even if all responses are inadequate, potentially leading to misleading results. Furthermore, the distribution of choices across these 4 options can provide rich insights into the relative strengths and weaknesses of each model and highlight areas where all models struggle (high “none” selections) or perform similarly well (high “neutral” selections). Ultimately, this method enhances evaluator confidence in their assessments because they are not forced into binary choices that may not reflect their true judgments.

### About IAA

From the full dataset, 100 cases were selected for IAA evaluation using random stratified sampling. This ensured proportional representation of all 4 task categories and coverage of multiple specialties. The sampled cases also included a mix of simple and complex examples, as well as shorter and longer inputs, to capture the diversity of the dataset while avoiding bias toward any specific task or medical domain.

In our study, we used a rigorous methodology to assess IAA among medical experts evaluating language model outputs. We used the ICC, specifically calculating ICC1, ICC2, ICC3, and their average measures counterparts ICC1k, ICC2k, and ICC3k [29].

ICCs are commonly denoted using a system that reflects both the statistical model and the reliability type being assessed. The notation ICC1, ICC2, and ICC3 refers to 1-way random effects, 2-way random effects, and 2-way mixed effects models, respectively. These are further specified with either “1” or “k” to indicate whether the reliability is for a single rater (1) or the average of k raters (k). For instance, ICC(2,1) represents a 2-way random effects model for single rater reliability, while ICC(3,k) indicates a 2-way mixed effects model for the average reliability of k raters.

ICC1 is used when subjects are assessed by different sets of randomly selected raters, ICC2 when all subjects are assessed by the same randomly selected raters, and ICC3 when the raters are the only ones of interest. This notation system allows researchers to precisely communicate the type of ICC used in their analyses, facilitating accurate interpretation and replication of results.

This comprehensive approach was applied to 3 distinct comparison scenarios: (1) MedS versus GPT-4o, (2) MedM versus GPT-4o, and (3) MedS versus MedM versus GPT-4o.

Our approach involved 3 qualified physicians independently annotating a seed dataset of 100 diverse medical queries, adhering to a predefined rubric focusing on factuality, clinical relevance, and conciseness. The ICC was selected over traditional methods such as Cohen κ or Spearman rank correlation due to its ability to handle multiple raters and continuous data, as well as its robustness across various data types and study designs.

The selection of multiple ICC forms (ICC1, ICC2, ICC3, ICC1k, ICC2k, and ICC3k) allowed us to account for different aspects of our study design:

- ICC1 and ICC1k: appropriate for scenarios where each subject is rated by different, randomly selected raters.
- ICC2 and ICC2k: suitable when the same set of randomly selected raters assesses all subjects.
- ICC3 and ICC3k: Used when a fixed set of raters evaluates all subjects. This comprehensive ICC analysis provided insights into both single-measure and average-measure reliability estimates, offering a nuanced understanding of the consistency in expert evaluations across different model comparisons.

The ICC’s statistical framework contributes to its accuracy in reflecting measurement scale reliability, particularly in handling missing data and varying sample sizes common in real-world research scenarios. Furthermore, its wide acceptance in health measurement, as recommended by the COSMIN (Consensus-Based Standards for the Selection of Health Measurement Instruments) guidelines, lends credibility to its use in medical research [30].

The results of this IAA analysis served as a crucial metric for validating the reliability of our annotation process and identifying areas for potential refinement in our evaluation methodology. This rigorous validation step is essential for ensuring the development of accurate and trustworthy AI models in health care applications, particularly in the context of NLP for medical information retrieval and dissemination.

### Washout Evaluation

To assess the temporal stability and intrarater reliability of expert annotations, we implemented a washout period analysis. This 2-phase annotation process involved a carefully selected 2-week interval between assessments, strategically chosen to minimize memory effects while ensuring consistent evaluation criteria. Expert annotators evaluated an identical set of questions at 2 distinct time points, without access to their previous responses during the second session. This design allowed us to quantify the consistency of individual annotators’ decisions over time.

To measure intrarater reliability, we calculated the percentage of unchanged responses for each annotator and used the ICC to account for agreement occurring by chance. This analysis facilitated the identification of potential ambiguities in our annotation schema and provided a robust measure of the temporal consistency of expert judgments, offering insights into the reproducibility of expert assessments and highlighting areas for potential refinement of evaluation criteria or additional annotator training.

### Ethical Considerations

This study did not involve human participants, animals, or any identifiable personal data. Therefore, institutional and local research ethics policies did not require ethics board approval for this type of study.

## Results

### Clinical Text Summarization

**Figure 1.**
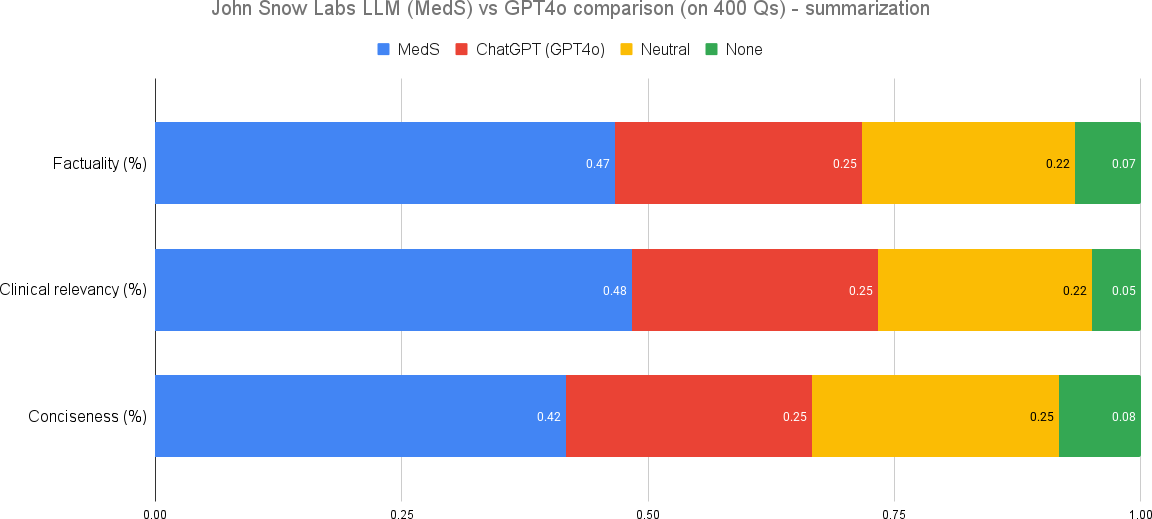
Clinical text summarization performance comparison. LLM: large language model; MedS: medical model–small; Q: question.

CS assessment helps ascertain whether these models can be trusted to support health care providers in high-stakes environments where errors can have significant consequences. Our comparative analysis of an 8 billion parameter model fine-tuned on medical data and GPT-4o revealed significant differences in performance across several key metrics, as illustrated in Figure 1.

- In terms of factuality, the MedS model was preferred 47% of the time, compared to 25% for GPT-4o, with the models being deemed equal in 22% of cases.
- Regarding clinical relevance, the MedS model was favored in 48% of instances, while GPT-4o was preferred in 25%.
- The MedS model also outperformed in conciseness, with a preference rate of 42% versus 25% for GPT-4o.

Evaluations were based on the models’ responses to specific medical summarization tasks. Sample questions posed to the models included: summarize the patient’s medical history and initial presentation, and summarize the final diagnosis of the lesion and the patient’s follow-up and recovery after surgery. These results suggest that domain-specific fine-tuning can lead to substantial improvements in performance, particularly in specialized fields such as medicine, even when compared to larger, more general models such as GPT-4o.

### Clinical Information Extraction

**Figure 2.**
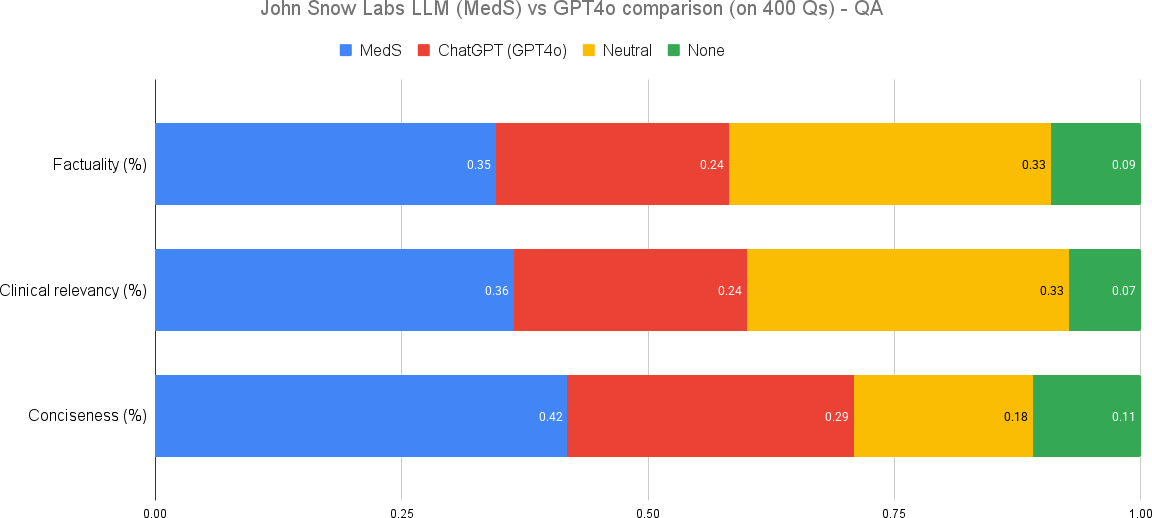
Clinical information extraction performance comparison. LLM: large language model; MedS: medical model–small; Q: question; QA: question answering.

Our analysis of an 8 billion parameter model fine-tuned on medical data compared to GPT-4o revealed notable performance differences, as illustrated in Figure 2.

- The MedS model exhibited a 10% advantage in factuality (35% vs 24%), with 33% of evaluations deemed neutral.
- In terms of relevance, the MedS model had a 12% advantage, while conciseness showed a 13% lead over GPT-4o.

### Biomedical QA

**Figure 3.**
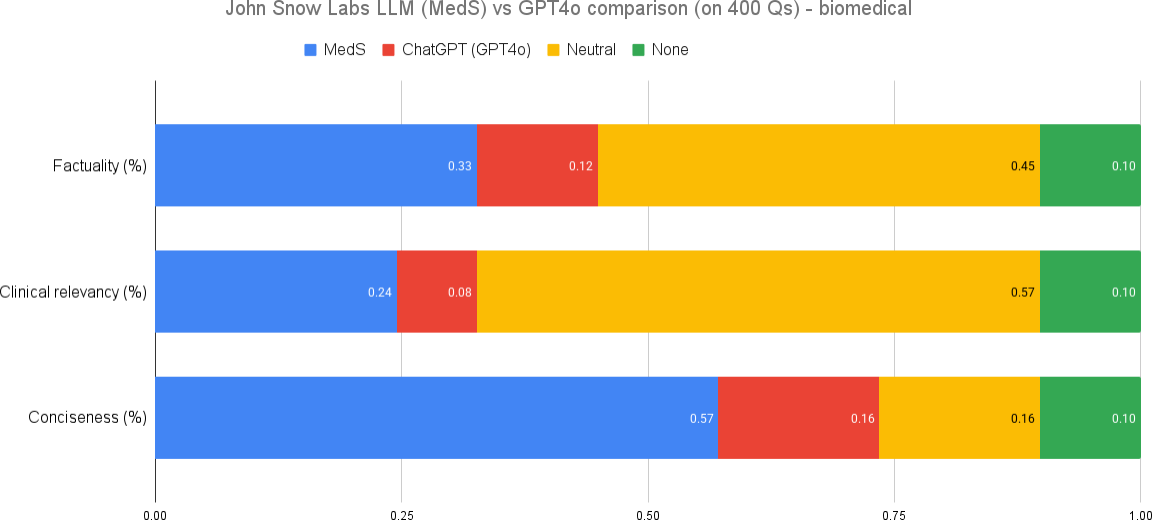
Biomedical question answering performance comparison. LLM: large language model; MedS: medical model–small; Q: question.

In biomedical research, the performance delta is significantly larger for our 8 billion parameter model, which has been fine-tuned on clinical and biomedical contexts as well as question-answering and summarization tasks, as demonstrated by Figure 3. Evaluations often involve providing an article or PubMed paper and asking specific questions or abstracting information directly from a short paragraph. In terms of factuality, MedS outperforms GPT-4o by a substantial margin. While clinical relevance assessments frequently yield neutral preferences—indicating similar performance between models—the enhanced training of our model enables it to excel in factual accuracy and contextually relevant tasks. This underscores the importance of specialized fine-tuning in achieving superior results in biomedical applications.

### Open-Ended Medical QA

**Figure 4.**
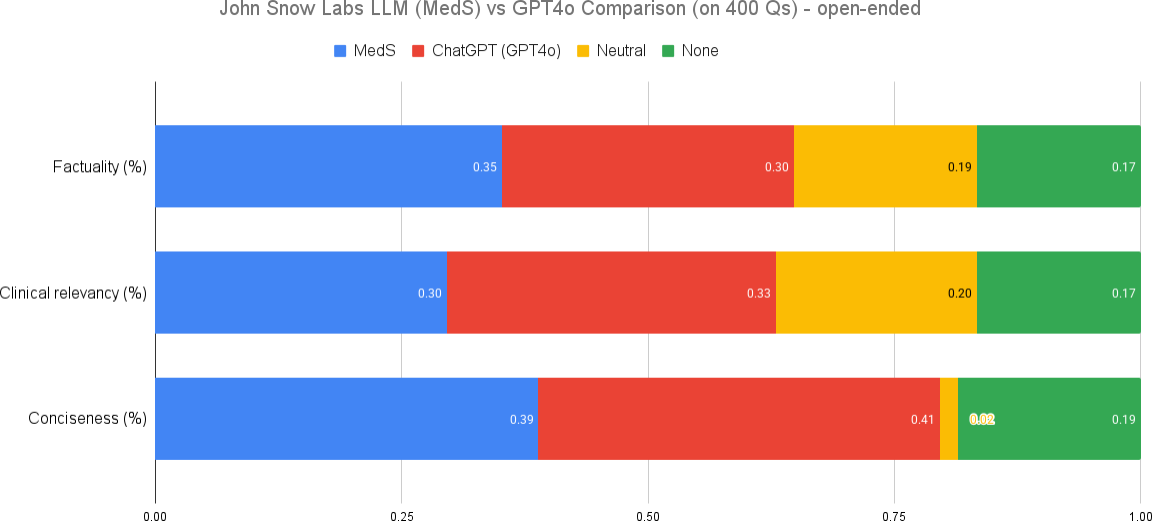
Open-ended medical question answering performance comparison. LLM: large language model; MedS: medical model–small; Q: question.

By assessing performance on open-ended QA tasks, researchers can evaluate a model’s overall medical knowledge and its readiness for real-world applications in clinical practice. In our evaluation of clinical relevance for open-ended medical questions without provided context, our 8 billion parameter model was unable to surpass GPT-4o’s performance. This outcome is understandable given the nature of the task and the models’ respective training. GPT-4o, being a more generalist model trained on a significantly larger dataset, has a broader knowledge base that proves advantageous in answering diverse medical questions without specific context. Our model, while excelling in targeted biomedical tasks, faces limitations in covering the entire spectrum of global medical knowledge. Despite this, it is noteworthy that our model still outperformed GPT-4o in factuality by 5 percentage points, highlighting its strength in accuracy within its specialized domain, as seen in Figure 4. This comparison underscores the trade-offs between specialized and generalist models, emphasizing the importance of context and task-specific training in AI model performance.

### LLM as a Judge

In our study, we used a novel evaluation framework incorporating both human expertise and LLM judgment. Specifically, in addition to 4 medical professionals, we used GPT-4o as a fifth evaluator.

For each test case, GPT-4o received the input clinical case or question, anonymized outputs from all candidate models (MedS, MedM, and GPT-4o), and the following instructions: “Rank the outputs based on factual accuracy, clinical relevance, and conciseness, from the perspective of a practicing physician who must make reliable decisions efficiently.” Conciseness was explicitly emphasized because, in real-world clinical settings, physicians need rapid, actionable summaries without redundant information. All models were treated identically, so GPT-4o could rank any model highest if it best satisfied these criteria.

Prioritizing conciseness reflects real clinical utility rather than introducing bias. Guiding the judge model with clinically meaningful criteria ensures the evaluation aligns with end user priorities. The preference observed for MedS indicates that, under these criteria, domain-specific models can generate outputs that better meet physician needs, even when evaluated by a larger general purpose model.

**Figure 5.**
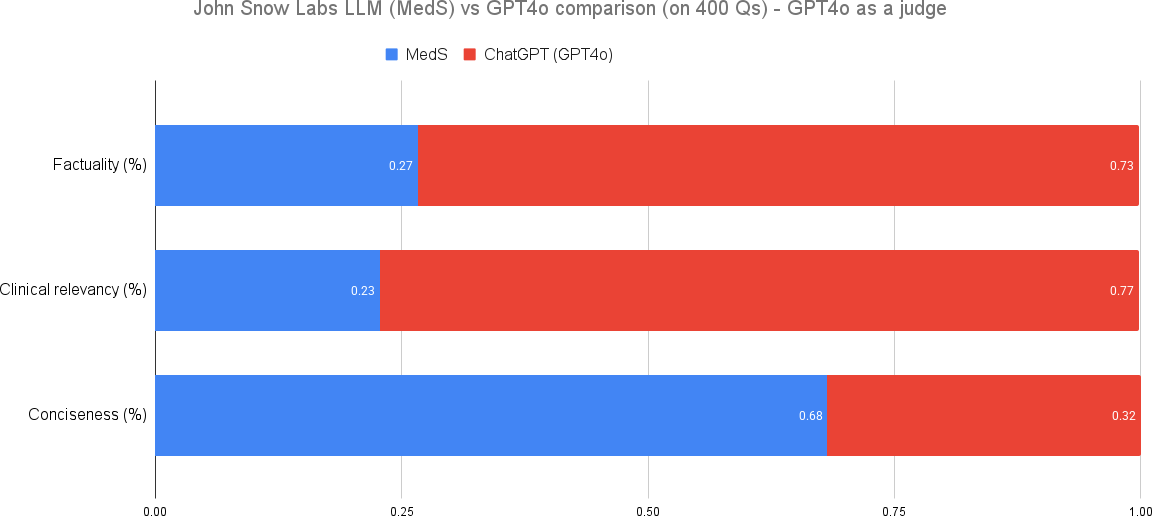
Performance comparison when using GPT-4o as a judge. LLM: large language model; MedS: medical model–small; Q: question.

As depicted in Figure 5, our findings revealed a significant discrepancy between GPT-4o’s ratings and those of the human reviewers when analyzed independently. We hypothesize that this divergence may be attributed to the LLM’s capacity to recognize its own generated responses, potentially leading to a favorable bias during the evaluation process. This observation aligns with recent research on LLM self-recognition and preference biases [31,32] and self-enhancement bias [33]. Our results underscore the importance of careful consideration when implementing LLM-based evaluation methods, particularly in domains requiring specialized knowledge, such as medicine.

### ICC Assessment

#### Overview

Table 1 ICC1 (absolute agreement) shows negative agreement because raters’ exact scores differ, and there is poor agreement on the absolute values of the ratings. In contrast, ICC2 and ICC3 (consistency-based agreement) show positive agreement because the raters are consistent in their rankings or pattern of ratings, even though exact values may be different. This suggests that raters may not give identical scores, but they tend to be consistent in their relative assessment of the targets. Therefore, consistency exists, despite consensus.

**Table 1.** Factuality over MedS^a^ versus GPT-4o: ICC^b^ results: ICC (assessing the reliability or agreement among raters). Please note that raters are consistent in their assessment, but do not agree with each other all the time.

Type	Description	ICC	*F* test (*df*)	*P* value	95% CI
ICC1	Single raters absolute	−0.095	0.739 (49, 1.0E+02)	.88	−0.22 to 0.07
ICC2	Single random raters	0.076	1.501 (49, 9.8E+01)	.045	−0.03 to 0.21
ICC3	Single fixed raters	0.143	1.501 (49, 9.8E+01)	.045	−0.02 to 0.33
ICC1k	Average raters absolute	−0.353	0.739 (49, 1.0E+02)	.88	−1.16 to 0.19
ICC2k	Average random raters	0.198	1.501 (49, 9.8E+01)	.045	−0.09 to 0.45
ICC3k	Average fixed raters	0.334	1.501 (49, 9.8E+01)	.045	−0.07 to 0.6

a MedS: medical model–small.

b ICC: intraclass correlation coefficient.

For ICC evaluation tables depicting reliability or agreement among raters for MedS versus GPT-4o, MedM versus GPT-4o, and MedS versus MedM versus GPT-4o please refer to Section S3 in Multimedia Appendix 1.

#### Factuality

This shows inconsistent reliability, with both negative and positive ICC values. The agreement improves somewhat with more raters, but the fluctuations suggest that the raters might not consistently agree on this dimension. When it comes to 3-way comparison, factuality also shows less variability and more consistency.

#### Clinical Relevance

Displays steady improvement in agreement as the number of raters increases, starting from poor reliability and reaching moderate levels. There is a clear upward trend, indicating that reliability improves with more raters.

#### Conciseness

The most reliable dimension, with ICC values indicating moderate to strong agreement among raters. The progression is steady and shows consistent improvement with more raters, suggesting that this dimension is easier to evaluate consistently. In general, conciseness appears to be the most reliable dimension, followed by clinical relevance, while factuality shows significant variability, making it the least reliable.

### Washout Period Analysis

#### Temporal Stability Assessment

Revisiting the same questions 2 weeks later allowed for an assessment of how consistently raters maintain their evaluations over time and whether they change their minds. The results indicate that annotators generally do not alter their assessments significantly during this period, suggesting a high level of stability and reliability in their evaluations, as depicted in Table 2. Additionally, the higher agreement observed in averaged ratings implies that collective evaluations are more consistent than those of individual raters. Among the participants, rater-1 demonstrated the highest reliability, exhibiting minimal changes in responses over the 2-week washout period. In contrast, rater-2 and rater-3 showed weaker agreement, with rater-3 being identified as the least consistent. Nevertheless, all raters exhibited statistically significant agreement, indicating that they largely maintained consistency in their answers over time. These findings underscore the importance of temporal stability in assessments and suggest that raters can provide reliable evaluations within a structured framework.

**Table 2.** ICC^a^ values and statistics per rater.

Type	Description	Rater 1	Rater 2	Rater 3
ICC1	Single raters absolute	0.927	0.653	0.601
ICC2	Single random raters	0.927	0.653	0.600
ICC3	Single fixed raters	0.928	0.657	0.596
ICC1k	Average raters absolute	0.962	0.790	0.751
ICC2k	Average random raters	0.962	0.790	0.750
ICC3k	Average fixed raters	0.963	0.793	0.747

a ICC: intraclass correlation coefficient.

#### Classification Report

As an alternative method, we assessed the consistency of raters across multiple evaluation rounds by treating the first evaluation as the ground truth. The analysis focused on how much raters changed their assessments in subsequent evaluations, with changes indicating lower *F*_1_-scores (Table 3). Rater-1 emerged as the most consistent overall, exhibiting high *F*_1_-scores and ICC values across most models and criteria, particularly demonstrating strong reliability in both conciseness and factuality. In contrast, rater-2 displayed greater variability, especially in factuality, where lower *F*_1_-scores indicated more frequent changes in judgment. However, rater-2 performed well in clinical relevance for certain models, suggesting that their consistency is criterion-dependent. Rater-3 also showed commendable performance, particularly in factuality, where their *F*_1_-scores were among the highest, consistent with the moderate to strong ICC values observed in the washout analysis. The findings indicate that factuality presents challenges for some raters—especially rater-2—while conciseness tends to be evaluated more consistently across all raters. The correlation between *F*_1_-scores and ICC values reinforces the reliability of these metrics as measures of rater consistency. This study highlights the importance of understanding how raters’ evaluations evolve and suggests that training or calibration may be beneficial, particularly for dimensions such as factuality, where variability is more pronounced. Future research should further explore the factors influencing rater decision-making and aim to enhance consistency across all evaluation criteria. This version emphasizes the classification aspect of your analysis while maintaining a scientific tone suitable for a paper.

**Table 3.** Rater *F*_1_-scores for different model comparisons: intrarater stability. We treated the first annotation round as the reference and compared each rater’s second-round labels against it. For each rater, we computed (1) the proportion of unchanged labels (identical labels in round-2 vs round-1) and (2) conventional classification metrics (precision, recall, and *F*_1_-score) where round-1 labels were treated as ground truth and round-2 as predictions.^a^

Comparison	Rater 1	Rater 2	Rater 3
MedS^b^ versus MedM^c^			
Clinical relevance	0.910	0.816	0.890
Conciseness	0.849	0.880	0.851
Factuality	0.901	0.756	0.875
MedS versus GPT-4o			
Clinical relevance	0.842	0.920	0.842
Conciseness	0.819	0.859	0.819
Factuality	0.862	0.859	0.814
MedM versus 3			
Clinical relevance	0.840	0.880	0.737
Conciseness	0.800	0.907	0.814
Factuality	0.849	0.859	0.746
Model 1 versus MedM versus GPT-4o			
Clinical relevance	0.826	0.809	0.812
Conciseness	0.824	0.772	0.860
Factuality	0.864	0.747	0.821

a The proportion unchanged is defined as the number of identical labels in both rounds divided by the total labels. Averaging across the 3 raters, the proportion unchanged was ≈0.83 (83%), implying ≈17% of labels differed between rounds; we conservatively reported this as “around 20%” in the main text. Importantly, *F*_1_-scores (reported in Section S5 in Multimedia Appendix 1) are a different statistic—they measure the harmonic mean of precision and recall for the target class when treating round-1 as ground truth—and can remain high (>0.8) even if a modest fraction of labels change, especially under class imbalance. We therefore report both measures (percent unchanged and *F*_1_-score) to provide a fuller picture of rater stability: percent-unchanged captures exact label repeatability, while *F*_1_-score captures reproduction of key classes (precision or recall performance).

b MedS: medical model–small.

c MedM: medical model–medium.

## Discussion

### Limitations and Future Work

#### Overview

While this study provides valuable insights into the reliability and consistency of language model evaluations, several limitations should be acknowledged, and potential avenues for future research identified.

#### Weighting of Evaluation Dimensions

The current study assigned equal weight to all rubrics (factuality, clinical relevance, and conciseness). However, in clinical applications, these dimensions may not be equally important. Factuality, for instance, may be of paramount importance in medical contexts, while conciseness might be considered less critical. Future studies should consider implementing a weighted scoring system that reflects the relative importance of each dimension in real-world applications.

#### Evaluator Bias Mitigation

Evaluators affiliated with the model-developing organization could introduce subconscious bias, so multiple safeguards were implemented. All medical experts were hired as external contractors and had no hand in model development, testing, or access to model outputs before the blinded evaluation study. The models evaluated were not publicly available, ensuring evaluators had no familiarity with their outputs. Though evaluators had worked with the organization before, all medical doctors were asked to blindly review all tasks, regardless of their specialty. Model outputs were anonymized and randomized, and evaluators received bias-awareness training focused on rubric-based scoring. IAA and ICC analyses ensured scoring consistency; a washout period helped measure intrarater reliability and minimize memory effects. These measures substantially reduced subconscious bias, supporting that evaluations reflected intrinsic model quality rather than organizational affiliation. Future research should consider fully independent audit teams for even greater credibility.

#### Medical Expertise and Specialization

The study revealed instances of numerous “neutral” responses, particularly in specialized medical areas. This suggests a potential limitation in the breadth of expertise among the evaluators. Future studies should consider expanding the evaluation panel to include a wider range of medical specialists, ensuring that each evaluated task is assessed by experts in the relevant field.

#### Evaluation Methodology

The current study used pairwise comparisons. While effective, this method may not capture the full spectrum of model performance. Future research could explore alternative methodologies, such as ranking-based evaluations, which might provide a more nuanced understanding of relative model performance across a broader range of tasks.

#### Time and Cost Considerations

The evaluation process, including the washout period, resulted in a significant time lag between model output and final assessment. Additionally, the use of human experts incurs substantial costs. Future research could explore methods to streamline the evaluation process and investigate cost-effective alternatives that maintain high reliability.

#### Model Training Bias

The superior performance of our models in conciseness may be attributed to specific training focusing on brevity. This highlights the importance of considering model training objectives when interpreting comparative results. Future studies should either ensure comparable training objectives across all models or explicitly account for these differences in the analysis.

#### Potential for AI-Assisted Evaluation

To mitigate human bias and potentially reduce costs, future research could explore the use of advanced language models as impartial judges. This approach would need to be carefully validated against human expert evaluations to ensure reliability and fairness.

#### Longitudinal Consistency

While this study included a washout period to assess short-term consistency, future research could benefit from longer-term longitudinal studies to evaluate the stability of assessments over extended periods and across different versions of the models.

#### Generalizability

The current study focused on specific models and medical contexts. Future research should aim to replicate these findings across a broader range of language models and diverse medical specialties to establish the generalizability of the evaluation framework.

#### Quantitative Analysis of Qualitative Feedback

In addition to numerical ratings, future studies could incorporate a more structured analysis of qualitative feedback from evaluators. This could provide deeper insights into the reasoning behind ratings and identify specific areas for model improvement.

#### Further Investigation

Factors influencing rater variability and strategies to enhance consistency among raters, particularly for those demonstrating lower reliability, should be investigated. A deeper understanding of rater dynamics over time and potential avenues for improving evaluation processes in various research contexts may be researched.

By addressing these limitations and exploring these future directions, subsequent research can further refine the methodology for evaluating medical language models, enhancing the reliability, objectivity, and practical applicability of such assessments in clinical settings.

### Conclusions

In our study, we aimed to bring a new methodology for benchmarking clinical LLMs. We observed that our methodology is effective, and as a positive side effect, we found that John Snow Labs’ health care–specific LLMs, trained on domain-specific data, outperformed GPT-4o. We evaluated our methods’ performance using these specialized LLMs as a test bed against GPT-4o on a range of medical tasks.

The results demonstrated that after fine-tuning on a domain-specific dataset, these specialized LLMs outperformed GPT-4o in the measured tasks. Notably, this superior performance was achieved using a model with 8 billion parameters, which can be deployed on-premise. This characteristic allows for private and efficient operation, addressing critical concerns in health care regarding data privacy and computational resource requirements. Moreover, the success of this small-sized model underscores a paradigm shift in model optimization, where targeted fine-tuning enables smaller models to rival and even surpass the performance of much larger generalist counterparts such as GPT-4o. Achieving a 5% advantage in factuality—a metric of paramount importance in medical applications—and outperforming GPT-4o by over 10% in conciseness demonstrates that strategic specialization can yield remarkable results. These findings challenge the assumption that scale alone dictates performance and highlight the potential of smaller, efficient models to deliver task-specific excellence in resource-constrained and privacy-sensitive domains.

The evaluation was conducted as a randomized, blind study, with medical doctors serving as evaluators. While interrater agreement was not strong, the evaluators demonstrated consistency in their observations across all questions. This methodology aligns with best practices in clinical research, emphasizing the importance of blinding in randomized trials assessing nonpharmacological treatments. Our assessment of LLM performance was conducted across multiple dimensions, including accuracy, correctness, and conciseness. This multifaceted approach acknowledged the complexity of medical information and the need for comprehensive evaluation metrics. The subjective nature of LLM evaluations underscores the importance of developing robust metrics, carefully crafted questions, and well-designed evaluation strategies. Furthermore, the evaluation process revealed that identifying certain errors required nuanced clinical knowledge, emphasizing the critical role of medical expertise in assessing AI models for health care applications.

This study encountered several challenges in the evaluation process, including issues with IAA, assessing fit for specific tasks, and determining model performance on unique data distributions. Additionally, the research team noted potential pitfalls in the analysis phase, particularly the risk of misattributing results if careful attention was not paid to model aliases and their corresponding data rows. This observation highlighted the importance of meticulous data management and analysis protocols in AI evaluation studies.

These findings contributed to the growing body of literature on the development and evaluation of AI models for health care applications. They underscored the potential of specialized LLMs in medical contexts while also highlighting the complexities and challenges inherent in their evaluation. Future research should focus on refining evaluation methodologies and metrics to ensure robust and clinically relevant assessments of AI models in health care.

## Supplementary material

10.2196/72153 Multimedia Appendix 1 Section S1: ICC interpretation, section S2: rater consistency, section S3: ICC statistics for raters, section S4: washout period statistics for raters, section S5: washout period statistics with classification report, section S6: sample model responses, section S7: comparing AI model responses, and section S8: prompt example. AI: artificial intelligence; ICC: intraclass correlation coefficient.

## Abbreviations

AI: artificial intelligence
CLEVER: Clinical Large Language Model Evaluation–Expert Review
COSMIN: Consensus-Based Standards for the Selection of Health Measurement Instruments
CS: clinical summarization
DR.BENCH: Diagnostic Reasoning Benchmark
ECFMG: Educational Commission for Foreign Medical Graduates
IAA: interannotator agreement
ICC: intraclass correlation coefficient
LLM: large language model
LoRA: Low-Rank Adaptation
MedM: medical model–medium
MedS: medical model–small
MMLU: Massive Multitask Language Understanding
NLP: natural language processing
QA: question answering
