# Supplementary material for: Clinical Large Language Model Evaluation by Expert Review (CLEVER): Framework Development and Validation
Source: JMIR AI. 2025 Dec 4;4:e72153. doi: 10.2196/72153 (PMC12677871; doi:10.2196/72153)
Supplement: Multimedia Appendix 1 [file ai-v4-e72153-s001.docx]

# Section S1: ICC interpretation

It is neither necessary nor practical to commit to a specific statistical model (one-way random, two- way random, or two-way mixed) at the outset of analyzing a matrix of single-score experimental data, such as in reliability studies. All three versions of the single-score intraclass correlation coefficient (ICC)—ICC(1) (classical), ICC(A,1) (absolute agreement), and ICC(C,1) (consistency)—can be computed and compared. When these coefficients show close agreement (within a few percent), it indicates a qualitative absence of systematic error (bias). Conversely, if ICC(C,1) is greater than ICC(A,1), this suggests the presence of bias. An F-test can be employed to assess whether the null hypothesis of no bias should be rejected; if it is not rejected, the ICC(1) value can be reported along with its confidence interval. However, if bias is present, ICC(1) becomes invalid and should not be utilized. Both ICC(A,1) and ICC(C,1) are valid regardless of whether measurement bias exists. These coefficients offer distinct but complementary perspectives on the reliability of the method and should be reported alongside their confidence intervals. Specifically, ICC(A,1) incorporates the influence of bias in its reliability estimate, while ICC(C,1) provides an estimate under the assumption that biases are absent [1].

ICC1k, ICC2k, and ICC3k enhance inter-rater reliability analysis by focusing on average ratings from multiple raters, with ICC1k applicable for different raters per subject, ICC2k for consistent randomly selected raters, and ICC3k for fixed rater groups, collectively offering a more comprehensive understanding of reliability in various research contexts.

The Intraclass Correlation Coefficient (ICC) values were interpreted using guidelines proposed by (Koo and Li, [2016),](#_bookmark15) which classify reliability as follows: poor (*<*0.50), moderate (0.50-0.75), good (0.75-0.90), and excellent (*>*0.90). This classification is more stringent than earlier guidelines, such as those by Cicchetti (1994), providing a more conservative assessment of reliability.

- **Overall Reliability:** The majority of ICC values fell within the excellent range (*>*0.90), indicating high reliability across all model comparisons and evaluation metrics (Factuality, Clinical Relevancy, and Conciseness).
- **Metric-Specific Patterns:** While all metrics showed high reliability, Conciseness consistently demonstrated slightly lower ICC values compared to Factuality and Clinical Relevancy. However, most Conciseness values still fell within the good to excellent range.
- **Model Comparison Variations:** The comparison involving all three models (MedS vs GPT4o vs MedM) exhibited slightly lower ICC values overall compared to pairwise comparisons. Nevertheless, these values remained within the good to excellent range.
- **Single vs. Average Measures:** Average measures (ICC1k, ICC2k, ICC3k) consistently demonstrated higher reliability compared to their corresponding single measures (ICC1, ICC2, ICC3), suggesting that using average ratings from multiple raters may provide more reliable assessments.

In assessing agreement and reliability among graders, we observed that for factuality, negative agreement indicated poor inter-rater agreement. In contrast, when evaluating consistency among raters, positive values were noted, signifying that while raters were consistent in their assessments, they did not necessarily agree with one another. Furthermore, when comparing dimensions across evaluations, it was evident that raters exhibited less consistency in factuality than in clinical relevance and conciseness.

# Section S2: Rater consistency

The ICC analysis reveals very high reliability in the assessments across all model comparisons and metrics. This robust reliability suggests that the evaluation methodology employed in this study provides consistent and dependable results for comparing the performance of different language models in terms of Factuality, Clinical Relevancy, and Conciseness.

The rater consistency analysis provides additional insights into the stability of judgments over time. While raters generally maintained consistent evaluations, the variability observed in Factuality ratings suggests that this dimension may be more susceptible to changes in perception or interpretation over time. Conversely, the high consistency in Conciseness ratings indicates greater confidence and stability in evaluations of this aspect.

These findings have important implications for the design and interpretation of language model evaluation studies. The high ICC values support the reliability of the assessment method, while the rater consistency analysis highlights areas where additional training or clearer evaluation criteria may be beneficial, particularly for the Factuality dimension.

## *Rater 1*

**Table S1.** Rater 1 ICC values and reliability grades for different model comparisons: rater 1 exhibited the highest consistency across all dimensions, with minimal variation in responses after the washout period, indicating a stable evaluation process.

| Comparison | Index | Factuality | Clinical Relevancy | Conciseness |
| --- | --- | --- | --- | --- |
|  | ICC1 | 0.970 (E) | 0.928 (E) | 0.882 (G) |
|  | ICC2 | 0.970 (E) | 0.928 (E) | 0.882 (G) |
| MedS vs GPT4o | ICC3 | 0.969 (E) | 0.928 (E) | 0.889 (G) |
|  | ICC1k | 0.985 (E) | 0.963 (E) | 0.937 (E) |
|  | ICC2k | 0.985 (E) | 0.963 (E) | 0.938 (E) |
|  | ICC3k | 0.984 (E) | 0.963 (E) | 0.941 (E) |
|  | ICC1 | 0.918 (E) | 0.940 (E) | 0.835 (G) |
|  | ICC2 | 0.918 (E) | 0.940 (E) | 0.835 (G) |
| MedM vs GPT4o | ICC3 | 0.920 (E) | 0.940 (E) | 0.836 (G) |
|  | ICC1k | 0.957 (E) | 0.969 (E) | 0.910 (E) |
|  | ICC2k | 0.958 (E) | 0.969 (E) | 0.910 (E) |
|  | ICC3k | 0.958 (E) | 0.969 (E) | 0.911 (E) |
|  | ICC1 | 0.956 (E) | 0.932 (E) | 0.868 (G) |
|  | ICC2 | 0.956 (E) | 0.932 (E) | 0.869 (G) |
| MedS vs MedM | ICC3 | 0.957 (E) | 0.931 (E) | 0.877 (G) |
|  | ICC1k | 0.977 (E) | 0.965 (E) | 0.929 (E) |
|  | ICC2k | 0.977 (E) | 0.965 (E) | 0.930 (E) |
|  | ICC3k | 0.978 (E) | 0.964 (E) | 0.934 (E) |
|  | ICC1 | 0.876 (G) | 0.856 (G) | 0.793 (G) |
|  | ICC2 | 0.876 (G) | 0.856 (G) | 0.793 (G) |
| MedS vs GPT4o vs MedM | ICC3 | 0.875 (G) | 0.858 (G) | 0.793 (G) |
|  | ICC1k | 0.934 (E) | 0.922 (E) | 0.885 (G) |
|  | ICC2k | 0.934 (E) | 0.922 (E) | 0.885 (G) |
|  | ICC3k | 0.933 (E) | 0.924 (E) | 0.885 (G) |

Note: M = Moderate reliability (0.5-0.75), G = Good reliability (0.75-0.9), E

= Excellent reliability (*>*0.9) [2]

## *Rater 2*

**Table S2.** Rater 2 ICC values and reliability grades for different model comparisons: rater 2 demonstrated good consistency, with only minor changes in ratings over time.

| Comparison | Index | Factuality | Clinical Relevancy | Conciseness |
| --- | --- | --- | --- | --- |
|  | ICC1 | 0.832 (G) | 0.845 (G) | 0.875 (G) |
|  | ICC2 | 0.833 (G) | 0.846 (G) | 0.875 (G) |
| MedS vs GPT4o | ICC3 | 0.843 (G) | 0.853 (G) | 0.875 (G) |
|  | ICC1k | 0.909 (E) | 0.916 (E) | 0.934 (E) |
|  | ICC2k | 0.909 (E) | 0.916 (E) | 0.934 (E) |
|  | ICC3k | 0.915 (E) | 0.921 (E) | 0.933 (E) |
|  | ICC1 | 0.569 (M) | 0.584 (M) | 0.738 (M) |
|  | ICC2 | 0.569 (M) | 0.583 (M) | 0.738 (M) |
| MedM vs GPT4o | ICC3 | 0.569 (M) | 0.579 (M) | 0.738 (M) |
|  | ICC1k | 0.726 (M) | 0.737 (M) | 0.850 (G) |
|  | ICC2k | 0.725 (M) | 0.736 (M) | 0.850 (G) |
|  | ICC3k | 0.725 (M) | 0.733 (M) | 0.849 (G) |
|  | ICC1 | 0.699 (M) | 0.816 (G) | 0.831 (G) |
|  | ICC2 | 0.698 (M) | 0.816 (G) | 0.831 (G) |
| MedS vs MedM | ICC3 | 0.697 (M) | 0.813 (G) | 0.829 (G) |
|  | ICC1k | 0.823 (G) | 0.899 (G) | 0.908 (E) |
|  | ICC2k | 0.822 (G) | 0.899 (G) | 0.908 (E) |
|  | ICC3k | 0.822 (G) | 0.897 (G) | 0.907 (E) |
|  | ICC1 | 0.737 (M) | 0.725 (M) | 0.802 (G) |
|  | ICC2 | 0.737 (M) | 0.725 (M) | 0.802 (G) |
| MedS vs GPT4o vs MedM | ICC3 | 0.736 (M) | 0.722 (M) | 0.799 (G) |
|  | ICC1k | 0.848 (G) | 0.841 (G) | 0.890 (G) |
|  | ICC2k | 0.848 (G) | 0.840 (G) | 0.890 (G) |
|  | ICC3k | 0.848 (G) | 0.838 (G) | 0.888 (G) |

Note: M = Moderate reliability (0.5-0.75), G = Good reliability (0.75-0.9), E

= Excellent reliability (*>*0.9) [2]

## *Rater 3*

**Table S3.** Rater 3 ICC values and reliability interpretation for different model comparisons: rater 3 showed the least consistency, particularly in Factuality, suggesting a higher likelihood of opinion changes after the two-week gap.

| Comparison | Index | Factuality | Clinical Relevancy | Conciseness |
| --- | --- | --- | --- | --- |
|  | ICC1 | 0.648 (M) | 0.754 (G) | 0.820 (G) |
|  | ICC2 | 0.647 (M) | 0.753 (G) | 0.820 (G) |
| MedS vs MedM | ICC3 | 0.645 (M) | 0.750 (G) | 0.817 (G) |
|  | ICC1k | 0.787 (G) | 0.860 (G) | 0.901 (E) |
|  | ICC2k | 0.786 (G) | 0.859 (G) | 0.901 (E) |
|  | ICC3k | 0.784 (G) | 0.857 (G) | 0.900 (E) |
|  | ICC1 | 0.778 (G) | 0.843 (G) | 0.845 (G) |
|  | ICC2 | 0.777 (G) | 0.843 (G) | 0.844 (G) |
| MedM vs GPT4o | ICC3 | 0.775 (G) | 0.846 (G) | 0.842 (G) |
|  | ICC1k | 0.875 (G) | 0.915 (E) | 0.916 (E) |
|  | ICC2k | 0.875 (G) | 0.915 (E) | 0.916 (E) |
|  | ICC3k | 0.873 (G) | 0.917 (E) | 0.914 (E) |
|  | ICC1 | 0.716 (M) | 0.756 (G) | 0.781 (G) |
|  | ICC2 | 0.716 (M) | 0.757 (G) | 0.782 (G) |
| MedS vs MedM | ICC3 | 0.712 (M) | 0.763 (G) | 0.783 (G) |
|  | ICC1k | 0.835 (G) | 0.861 (G) | 0.877 (G) |
|  | ICC2k | 0.834 (G) | 0.862 (G) | 0.877 (G) |
|  | ICC3k | 0.832 (G) | 0.866 (G) | 0.878 (G) |
|  | ICC1 | 0.535 (M) | 0.764 (G) | 0.689 (M) |
|  | ICC2 | 0.533 (M) | 0.764 (G) | 0.694 (M) |
| MedS vs GPT4o vs MedM | ICC3 | 0.529 (M) | 0.761 (G) | 0.715 (M) |
|  | ICC1k | 0.697 (M) | 0.866 (G) | 0.816 (G) |
|  | ICC2k | 0.696 (M) | 0.866 (G) | 0.819 (G) |
|  | ICC3k | 0.692 (M) | 0.864 (G) | 0.834 (G) |

Note: M = Moderate reliability (0.5-0.75), G = Good reliability (0.75-0.9), E

= Excellent reliability (*>*0.9) [2]

# Section S3: ICC statistics for raters

The analysis of inter-rater reliability across three dimensions reveals distinct patterns: Factuality exhibits inconsistent reliability, characterized by both negative and positive ICC values, with improved agreement as the number of raters increases but notable fluctuations; Clinical Relevancy shows a steady improvement in agreement from poor to moderate reliability, indicating that more raters enhance assessment consistency; and Conciseness stands out as the most reliable dimension, demonstrating moderate to strong agreement among raters and consistent improvement with additional raters, suggesting it is easier to evaluate consistently.

## *MedS vs GPT4o ICC values for different metrics*

**Table S4.** MedS vs GPT4o ICC values for different metrics.

| **Metric** | **Factuality** | **Clinical Relevance** | **Conciseness** |
| --- | --- | --- | --- |
| ICC1 | -0.095 | 0.090 | 0.229 |
| ICC2 | 0.076 | 0.169 | 0.263 |
| ICC3 | 0.143 | 0.228 | 0.303 |
| ICC1k | -0.353 | 0.228 | 0.471 |
| ICC2k | 0.198 | 0.378 | 0.517 |
| ICC3k | 0.334 | 0.469 | 0.566 |

## *MedM vs GPT4o ICC values for different metrics*

**Table S5.** MedM vs GPT4o ICC values for different metrics.

| **Metric** | **Factuality** | **Clinical Relevance** | **Conciseness** |
| --- | --- | --- | --- |
| ICC1 | -0.157 | 0.162 | 0.173 |
| ICC2 | 0.020 | 0.217 | 0.215 |
| ICC3 | 0.037 | 0.270 | 0.253 |
| ICC1k | -0.686 | 0.366 | 0.386 |
| ICC2k | 0.057 | 0.453 | 0.451 |
| ICC3k | 0.102 | 0.526 | 0.504 |

## *MedS vs MedM vs GPT4o ICC values for different metrics*

**Table S6.** MedS vs MedM vs GPT4o ICC values for different metrics.

| **Metric** | **Factuality** | **Clinical Relevance** | **Conciseness** |
| --- | --- | --- | --- |
| ICC1 | 0.260 | 0.309 | 0.418 |
| ICC2 | 0.270 | 0.313 | 0.416 |
| ICC3 | 0.281 | 0.318 | 0.412 |
| ICC1k | 0.514 | 0.573 | 0.683 |
| ICC2k | 0.526 | 0.578 | 0.681 |
| ICC3k | 0.540 | 0.584 | 0.678 |

# Section S4: Washout period statistics for raters

A washout period analysis involved asking the same questions to the same raters with a two-week interval between assessments, allowing us to measure the stability of raters’ judgments and determine whether they significantly change their minds over the washout period.

## *Rater 1*

Rater demonstrated the highest level of reliability in between raters, with minimal changes in responses over the washout period. This consistency suggests that rater’s assessments are highly stable and dependable over time. Rater’s judgments are particularly robust and less susceptible to temporal variations.

**Table S7.** Rater-1 ICC values and statistics.

| Type | Description | ICC | F | df1 | df2 | p-value | 95% CI |
| --- | --- | --- | --- | --- | --- | --- | --- |
| ICC1 | Single raters absolute | 0.927 | 26.451 | 49 | 50 | 3.338e-23 | [0.88, 0.96] |
| ICC2 | Single random raters | 0.927 | 26.958 | 49 | 49 | 5.103e-23 | [0.88, 0.96] |
| ICC3 | Single fixed raters | 0.928 | 26.958 | 49 | 49 | 5.103e-23 | [0.88, 0.96] |
| ICC1k | Average raters absolute | 0.962 | 26.451 | 49 | 50 | 3.338e-23 | [0.93, 0.98] |
| ICC2k | Average random raters | 0.962 | 26.958 | 49 | 49 | 5.103e-23 | [0.93, 0.98] |
| ICC3k | Average fixed raters | 0.963 | 26.958 | 49 | 49 | 5.103e-23 | [0.93, 0.98] |

## *Rater 2*

Rater exhibited weaker agreement compared to Rater-1, indicating some variability in assessments over the two-week period. Despite this lower consistency, rater still showed statistically significant agreement between the two time points. The moderate level of agreement suggests that while rater’s judgments are generally reliable, there is still room for fluctuation in their assessments over time.

**Table S8.** Rater-2 ICC values and statistics.

| Type | Description | ICC | F | df1 | df2 | p-value | 95% CI |
| --- | --- | --- | --- | --- | --- | --- | --- |
| ICC1 | Single raters absolute | 0.653 | 4.758 | 49 | 50 | 8.494e-08 | [0.46, 0.79] |
| ICC2 | Single random raters | 0.653 | 4.823 | 49 | 49 | 8.428e-08 | [0.46, 0.79] |
| ICC3 | Single fixed raters | 0.657 | 4.823 | 49 | 49 | 8.428e-08 | [0.46, 0.79] |
| ICC1k | Average raters absolute | 0.790 | 4.758 | 49 | 50 | 8.494e-08 | [0.63, 0.88] |
| ICC2k | Average random raters | 0.790 | 4.823 | 49 | 49 | 8.428e-08 | [0.63, 0.88] |
| ICC3k | Average fixed raters | 0.793 | 4.823 | 49 | 49 | 8.428e-08 | [0.63, 0.88] |

## *Rater 3*

Among the three raters, rater demonstrated the least consistency over the washout period. However, despite being the least consistent, rater still showed statistically significant agreement between the initial and follow-up assessments. This suggests that while Rater-3’s judgments may be more susceptible to change over time, there is still a meaningful level of reliability in their assessments.

**Table S9.** Rater-3: ICC values and statistics.

| Type | Description | ICC | F | df1 | df2 | p-value | 95% CI |
| --- | --- | --- | --- | --- | --- | --- | --- |
| ICC1 | Single raters absolute | 0.601 | 4.010 | 60 | 61 | 1.031e-07 | [0.41, 0.74] |
| ICC2 | Single random raters | 0.600 | 3.947 | 60 | 60 | 1.627e-07 | [0.41, 0.74] |
| ICC3 | Single fixed raters | 0.596 | 3.947 | 60 | 60 | 1.627e-07 | [0.41, 0.74] |
| ICC1k | Average raters absolute | 0.751 | 4.010 | 60 | 61 | 1.031e-07 | [0.59, 0.85] |
| ICC2k | Average random raters | 0.750 | 3.947 | 60 | 60 | 1.627e-07 | [0.58, 0.85] |
| ICC3k | Average fixed raters | 0.747 | 3.947 | 60 | 60 | 1.627e-07 | [0.58, 0.85] |

# Section S5: Washout period statistics with classification report

If we consider one of the rounds as a ground truth, we can calculate a classification metrics and use that as a yardstick for a comparison.

**Table S10.** ICC Values for different model comparisons and raters. Note: percent-changed (label repeatability) and F1 (precision/recall) are distinct; both are reported.

| Type | Description | Rater 1 | Rater 2 | Rater 3 |
| --- | --- | --- | --- | --- |
| ICC1 | Single raters absolute | 0.927 | 0.653 | 0.601 |
| ICC2 | Single random raters | 0.927 | 0.653 | 0.600 |
| ICC3 | Single fixed raters | 0.928 | 0.657 | 0.596 |
| ICC1k | Average raters absolute | 0.962 | 0.790 | 0.751 |
| ICC2k | Average random raters | 0.962 | 0.790 | 0.750 |
| ICC3k | Average fixed raters | 0.963 | 0.793 | 0.747 |

**Table 2.** ICC Values and Statistics per Rater

| Comparison | Metric | Rater-1 | Rater-2 | Rater-3 |
| --- | --- | --- | --- | --- |
|  | ClinicalRelevancy | 0.862 | 0.886 | 0.861 |
| MedS vs MedM | Conciseness | 0.879 | 0.825 | 0.857 |
|  | Factuality | 0.910 | 0.868 | 0.801 |
|  | ClinicalRelevancy | 0.891 | 0.893 | 0.857 |
| MedM vs GPT4o | Conciseness | 0.860 | 0.861 | 0.869 |
|  | Factuality | 0.901 | 0.798 | 0.793 |
|  | ClinicalRelevancy | 0.840 | 0.708 | 0.900 |
| MedS vs MedM | Conciseness | 0.729 | 0.810 | 0.920 |
|  | Factuality | 0.798 | 0.788 | 0.879 |
|  | ClinicalRelevancy | 0.824 | 0.812 | 0.811 |
| MedS vs GPT4o vs MedM | Conciseness | 0.817 | 0.861 | 0.769 |
|  | Factuality | 0.864 | 0.826 | 0.749 |

# Section S6: Sample model responses

## *SME IDE*

Tables in this section depict model task, prompt and context as seen and evaluated by raters. At each step, model order is shuffled in order to mitigate potential biases that may arise from the order in which models are presented.

**Table S11.** Summarization task sample: models are evaluated in four different task categories. Table presents an example for summarization task.

| **Category** | **Prompt** | **Text** |
| --- | --- | --- |
| Summarization | Summarize the treatment pro-  tocol for each study arm from the given text: | Background: Globally, head and neck squamous  cell carcinoma (HNSCC) is the seventh most common malignancy. Despite aggressive multi- modal treatment approaches, recurrent and/or metastatic (R/M) disease develops in *<*50% of patients. In this setting, pembrolizumab was ap- proved for patients with PD-L1 expression. How- ever, response rates with checkpoint inhibitor monotherapy remain limited and strategies to strengthen tumor-directed immune responses are needed.  Objective: The FOCUS trial is designed to esti- mate the effectiveness of UV1 vaccination in combi- nation with pembrolizumab versus pembrolizumab as a single agent in patients with R/M HNSCC. Methods and analysis: The FOCUS trial is a two- armed, randomized, multicenter phase II study which was designed to evaluate the efficacy and fea- sibility of the hTERT-targeted cancer vaccine UV1 as add-on to pembrolizumab in the 1st line treat- ment of patients with R/M PD-L1 . . . *(truncated)* |

**Table S12.** User IDE: for each question, model numerations are shuffled. Rater selects between given model numbers, or opts for neutral or none.

|  | **model-1 vs model-2** | **model-1 vs model-3** | **model-2 vs model-3** |
| --- | --- | --- | --- |
| Factuality | Neutral  model-1 model-1 | Neutral  model-1 model-1 | Neutral  model-2 model-2 |
| Clinical Relevancy | Neutral  model-1 model-1 | Neutral  model-1 model-1 | Neutral  model-2 model-2 |
| Conciseness | Neutral  Neutral model-1 | model-3  Neutral model-1 | model-3  Neutral model-2 |

## *Model Responses*

Boxes below display sample responses from different models in our blind randomized experiment. This allows raters to evaluate and compare the outputs without knowledge of their source, ensuring an unbiased assessment of each model’s performance across various criteria.

Model 1

Model 1

The treatment protocol for each study arm is as follows: Arm A (pembrolizumab monotherapy):

- Patients receive pembrolizumab at 200mg flat dose iv every 3 weeks.
- Administration starts at week 1.
- The duration of treatment will be 12 weeks. Arm B (pembrolizumab + UVI vaccination):
- Patients receive pembrolizumab at 200mg iv every 3 weeks in combination with LJVI vaccination (300pg IJVI plus 75pg GM-CSF as adjuvant).
- Three OVI vaccinations are applied during the week before initiation of pembrolizumab.
- Five vaccinations are applied every 3 weeks on dl of each cycle (5 cycles in total).
- Administration of pembrolizumab starts at week 2.
- The duration of treatment will be 13 weeks.

Note: All patients will receive pembrolizumab until disease progression and up to two years in both arms.

The treatment protocol for each study arm is as follows: Arm A (pembrolizumab monotherapy):

- Patients receive pembrolizumab at 200mg flat dose iv every 3 weeks.
- Administration starts at week 1.
- The duration of treatment will be 12 weeks. Arm B (pembrolizumab + UVI vaccination):
- Patients receive pembrolizumab at 200mg iv every 3 weeks in combination with LJVI vaccination (300pg IJVI plus 75pg GM-CSF as adjuvant).
- Three OVI vaccinations are applied during the week before initiation of pembrolizumab.
- Five vaccinations are applied every 3 weeks on dl of each cycle (5 cycles in total).
- Administration of pembrolizumab starts at week 2.
- The duration of treatment will be 13 weeks.

Note: All patients will receive pembrolizumab until disease progression and up to two years in both arms.

Model 2

Model 2

The FOCUS trial is a randomized, multicenter phase II study designed to evaluate the efficacy and feasibility of the hTERT-targeted cancer vaccine LJVI as an add-on to pembrolizumab in the first-line treatment of patients with recurrent and/or metastatic (RIM) PD-L1 positive head and neck squamous cell carcinoma (HNSCC). There are two study arms in the FOCUS trial:

- Arm A: Patients receive pembrolizumab at 200mg flat dose iv every 3 weeks.
- Administration starts at week 1.
- The duration of treatment will be 12 weeks.
- Arm B

The FOCUS trial is a randomized, multicenter phase II study designed to evaluate the efficacy and feasibility of the hTERT-targeted cancer vaccine LJVI as an add-on to pembrolizumab in the first-line treatment of patients with recurrent and/or metastatic (RIM) PD-L1 positive head and neck squamous cell carcinoma (HNSCC). There are two study arms in the FOCUS trial:

- Arm A: Patients receive pembrolizumab at 200mg flat dose iv every 3 weeks.
- Administration starts at week 1.
- The duration of treatment will be 12 weeks.
- Arm B

Model 3

Model 3

The FOCUS trial involves two study arms to treat patients with recurrent and/or metastatic head and neck squamous cell carcinoma (RIM HNSCC) who express PD-L1 (combined positive score *>*= 21). Here is a summary of the treatment protocols for each arm: Arm A:

- Treatment Regimen: Pembrolizumab: 200 mg flat dose administered intravenously

(IV) every three weeks.

- Start of Administration: Week 1.
- Duration of Treatment: Until disease progression or up to two years, spanning a duration of 12 weeks for the initial phase.

Arm B:

- Treatment Regimen:

The FOCUS trial involves two study arms to treat patients with recurrent and/or metastatic head and neck squamous cell carcinoma (RIM HNSCC) who express PD-L1 (combined positive score *>*= 21). Here is a summary of the treatment protocols for each arm: Arm A:

- Treatment Regimen: Pembrolizumab: 200 mg flat dose administered intravenously

(IV) every three weeks.

- Start of Administration: Week 1.
- Duration of Treatment: Until disease progression or up to two years, spanning a duration of 12 weeks for the initial phase.

Arm B:

- Treatment Regimen:

# Section S7: Comparing AI model responses

This appendix presents examples comparing responses from different AI models, highlighting the preferred responses and reasons for preference, as well as portraying prompted questions.

**Example 1: Barrett’s Esophagus Management**

**Given:** Patient note with multiple conditions including Barrett’s esophagus.

**Question:** Given the note, how is the Barrett’s esophagus managed in this patient?

**Preferred response (MedS):** Barrett’s esophagus is managed in this patient by continuing omeprazole and following up with a gastroenterologist (GI).

**GPT-4o response:** Based on the provided note, the management of Barrett’s esophagus in this patient involves:

- Follow-up with a Gastroenterologist: This indicates that the patient needs to be monitored by a specialist for any changes or progression of the disease.
- Continue omeprazole: This medication is likely being used to reduce acid reflux, which is a common cause of Barrett’s esophagus. The patient will need to continue taking omeprazole as directed by their healthcare provider.

**Why MedS is preferred:** The MedS model concisely answers the question in the first sentence, quickly giving the answer. GPT-4o redundantly repeats part of the prompt and gives an answer more suited for general population rather than medical specialists.

**Example 1: Barrett’s Esophagus Management**

**Given:** Patient note with multiple conditions including Barrett’s esophagus.

**Question:** Given the note, how is the Barrett’s esophagus managed in this patient?

**Preferred response (MedS):** Barrett’s esophagus is managed in this patient by continuing omeprazole and following up with a gastroenterologist (GI).

**GPT-4o response:** Based on the provided note, the management of Barrett’s esophagus in this patient involves:

- Follow-up with a Gastroenterologist: This indicates that the patient needs to be monitored by a specialist for any changes or progression of the disease.
- Continue omeprazole: This medication is likely being used to reduce acid reflux, which is a common cause of Barrett’s esophagus. The patient will need to continue taking omeprazole as directed by their healthcare provider.

**Why MedS is preferred:** The MedS model concisely answers the question in the first sentence, quickly giving the answer. GPT-4o redundantly repeats part of the prompt and gives an answer more suited for general population rather than medical specialists.

**Example 2: TGF-***β* **Superfamily Proteins**

**Given:** Text about TGF-*β* signaling pathway and related proteins.

**Question:** Given the text, which proteins belong to TGF-*β* superfamily?

**Preferred response (MedS):** TGF-*β* superfamily proteins mainly include TGF-*β*s, activins, BMPs, growth and differentiation factors, and cofactors/adaptors.

**GPT4o response:** The TGF-*β* (Transforming Growth Factor-beta) superfamily of proteins includes:

- TGF-*β*s (Transforming Growth Factors-beta)
- Activins
- BMPs (Bone Morphogenetic Proteins)
- Growth and Differentiation Factors (additional proteins categorized under this term)
- Cofactors/Adaptors

**Why MedS is preferred:** MedS gave a direct answer with a shorter text format, while GPT4o answered correctly but included additional information that wasn’t requested.

**Example 2: TGF-***β* **Superfamily Proteins**

**Given:** Text about TGF-*β* signaling pathway and related proteins.

**Question:** Given the text, which proteins belong to TGF-*β* superfamily?

**Preferred response (MedS):** TGF-*β* superfamily proteins mainly include TGF-*β*s, activins, BMPs, growth and differentiation factors, and cofactors/adaptors.

**GPT4o response:** The TGF-*β* (Transforming Growth Factor-beta) superfamily of proteins includes:

- TGF-*β*s (Transforming Growth Factors-beta)
- Activins
- BMPs (Bone Morphogenetic Proteins)
- Growth and Differentiation Factors (additional proteins categorized under this term)
- Cofactors/Adaptors

**Why MedS is preferred:** MedS gave a direct answer with a shorter text format, while GPT4o answered correctly but included additional information that wasn’t requested.

**Example 3: Intraoperative PTH Level**

**Given:** Case description of a 62-year-old woman with primary hyperparathyroidism.

**Question:** Given the text, what was the intraoperative PTH level of the patient?

**Preferred response (MedS):** The intraoperative PTH level of the patient was 83 pg/ml at baseline and 85 pg/ml at the close of the surgery.

**GPT4o response:** The intraoperative PTH level of the patient did not decline significantly during the initial surgery, remaining at 85 pg/ml at the close of the surgery, compared to a baseline of 83 pg/ml. During the subsequent trans-oral robotic parathyroidectomy in November 2022, the baseline PTH was reported to be 158 pg/ml, which dropped to 110 pg/ml within 10 minutes post-surgery.

**Why MedS is preferred:** MedS model successfully reported the requested information, whereas GPT didn’t answer the question directly and gave an irrelevant response.

**Example 3: Intraoperative PTH Level**

**Given:** Case description of a 62-year-old woman with primary hyperparathyroidism.

**Question:** Given the text, what was the intraoperative PTH level of the patient?

**Preferred response (MedS):** The intraoperative PTH level of the patient was 83 pg/ml at baseline and 85 pg/ml at the close of the surgery.

**GPT4o response:** The intraoperative PTH level of the patient did not decline significantly during the initial surgery, remaining at 85 pg/ml at the close of the surgery, compared to a baseline of 83 pg/ml. During the subsequent trans-oral robotic parathyroidectomy in November 2022, the baseline PTH was reported to be 158 pg/ml, which dropped to 110 pg/ml within 10 minutes post-surgery.

**Why MedS is preferred:** MedS model successfully reported the requested information, whereas GPT didn’t answer the question directly and gave an irrelevant response.

**Example 4: First-line Treatment for Classic Hodgkin’s Lymphoma Question:** What is the first-line treatment for Classic Hodgkin’s lymphoma?

**Preferred response (MedS):** The first-line treatment for Classic Hodgkin’s lymphoma typically involves chemotherapy. One of the most common chemotherapy regimens used is ABVD, which stands for:

- **A**driamycin (also known as doxorubicin)
- **B**leomycin
- **V**inblastine
- **D**acarbazine

**GPT4o response:** The first-line treatment for Classic Hodgkin’s lymphoma typically involves a combination of chemotherapy and radiation therapy.

**Why MedS is preferred:** MedS model gave a correct answer by including the specific chemotherapy regimen info, whereas GPT answered the question in a very general way.

**Example 4: First-line Treatment for Classic Hodgkin’s Lymphoma Question:** What is the first-line treatment for Classic Hodgkin’s lymphoma?

**Preferred response (MedS):** The first-line treatment for Classic Hodgkin’s lymphoma typically involves chemotherapy. One of the most common chemotherapy regimens used is ABVD, which stands for:

- **A**driamycin (also known as doxorubicin)
- **B**leomycin
- **V**inblastine
- **D**acarbazine

**GPT4o response:** The first-line treatment for Classic Hodgkin’s lymphoma typically involves a combination of chemotherapy and radiation therapy.

**Why MedS is preferred:** MedS model gave a correct answer by including the specific chemotherapy regimen info, whereas GPT answered the question in a very general way.

# Section S8: Prompt example

## *Prompt Example Code*

The following prompt example was used to elicit responses from the language model and is presented here for reference and reproducibility. For increased determinism temperature value has been set to 0 and top_p value has been set to 1.

system_prompt = "You are a helpful medical assistant. Provide accurate, evidence-based information in response to the following question. Organize the response with clear hierarchical headings and include a conclusion if necessary."

user_prompt = f"""Answer the question given the following content:

Question: {question}

Content: {content}

"""

#Question and content placeholders come from our (500) Question Excel Sheet

def generate_response(user_prompt,system_prompt):
    messages = []

    if system_prompt:
        messages.append({
            "role": "system",
            "content": system_prompt
        })

    messages.append({
        "role": "user",
        "content": user_prompt
    })

    outputs = model.chat(messages, sampling_params=sampling_params)

    response = outputs[0].outputs[0].text

    response = "\n".join(line for line in response.splitlines() if line.strip())

    return response

## L *Prompt Examples*

Summarize the following text

Summarize the patient's medical history from the following text:

Summarize the patient's follow-up care from the following text:

Summarize the initial diagnostic findings from the following text:

Summarization

What is the relationship between dystonia and myoclonus in corticobasal syndromepatients?

What is the alien-limb syndrome, and how is it categorized?

Open Ended

Given the note, what are the mechanisms behind ALDH functions?

Given the article, what is sNFL used for?

Given the report, what biomarkers are commonly negative in APL cases?

Biomedical QA

Given the medical note, what were the conclusions of the study?

Given the article, what was the target diagnosis in this study?

Given the note, did all the patients have pathological examination?

QA

# References

1. Liljequist D, Elfving B, Skavberg Roaldsen K. Intraclass correlation - a discussion and demonstration of basic features. PLoS ONE. 2019;14(7):e0219854. [doi: 10.1371/journal.pone.0219854] [Medline: 31329615]

2. Koo TK, Li MY. A guideline of selecting and reporting intraclass correlation coefficients for reliability reseGuideline of Selecting and Reporting Intraclass Correlation Coefficients for Reliability Research. J Chiropr Med. Jun 2016;15(2):155-163. [doi: 10.1016/j.jcm.2016.02.012] [Medline: 27330520]
